# Semantic retrieval during overt picture description: Left anterior temporal or the parietal lobe?

**DOI:** 10.1016/j.neuropsychologia.2014.12.012

**Published:** 2015-09

**Authors:** Fatemeh Geranmayeh, Robert Leech, Richard J.S. Wise

**Affiliations:** Computational Cognitive and Clinical Neuroimaging Laboratory, Imperial College, Hammersmith Hospital, London W12 0NN, UK

**Keywords:** Semantic, Anterior temporal lobe

## Abstract

Retrieval of semantic representations is a central process during overt speech production. There is an increasing consensus that an amodal semantic ‘hub’ must exist that draws together modality-specific representations of concepts. Based on the distribution of atrophy and the behavioral deficit of patients with the semantic variant of fronto-temporal lobar degeneration, it has been proposed that this hub is localized within both anterior temporal lobes (ATL), and is functionally connected with verbal ‘output’ systems via the left ATL. An alternative view, dating from Geschwind's proposal in 1965, is that the angular gyrus (AG) is central to object-based semantic representations. In this fMRI study we examined the connectivity of the left ATL and parietal lobe (PL) with whole brain networks known to be activated during overt picture description. We decomposed each of these two brain volumes into 15 regions of interest (ROIs), using independent component analysis. A dual regression analysis was used to establish the connectivity of each ROI with whole brain-networks. An ROI within the left anterior superior temporal sulcus (antSTS) was functionally connected to other parts of the left ATL, including anterior ventromedial left temporal cortex (partially attenuated by signal loss due to susceptibility artifact), a large left dorsolateral prefrontal region (including ‘classic’ Broca's area), extensive bilateral sensory-motor cortices, and the length of both superior temporal gyri. The time-course of this functionally connected network was associated with picture description but not with non-semantic baseline tasks. This system has the distribution expected for the production of overt speech with appropriate semantic content, and the auditory monitoring of the overt speech output. In contrast, the only left PL ROI that showed connectivity with brain systems most strongly activated by the picture-description task, was in the superior parietal lobe (supPL). This region showed connectivity with predominantly posterior cortical regions required for the visual processing of the pictorial stimuli, with additional connectivity to the dorsal left AG and a small component of the left inferior frontal gyrus. None of the other PL ROIs that included part of the left AG were activated by Speech alone. The best interpretation of these results is that the left antSTS connects the proposed semantic hub (specifically localized to ventral anterior temporal cortex based on clinical neuropsychological studies) to posterior frontal regions and sensory-motor cortices responsible for the overt production of speech.

## Highlights

• Semantic cognition depends on connectivity of semantic hub with input/output systems.
• We examined the connectivity of the left ATL and PL sub-regions during speech.
• An ATL sub-region was linked to Broca's area and sensory-motor cortices during Speech.
• A PL subregion was linked to systems involved in predominantly perceptual processing of picture stimuli.
• The left ATL connects the proposed semantic hub to output systems for overt speech production.

## 1 Introduction

Spoken language comprehension and production are dependent on widely distributed sensory, motor and linguistic systems comprising functionally specialized components (Indefrey and Levelt, 2004; Price, 2012). However, language is nothing without access to semantic representations. Geschwind (1965a, 1965b) proposed that the expansion of the human parietal lobe relative to that of other primates and mammalian species, its polysensory connections, its independence from the limbic system, and its anatomical connections with Wernicke's area in the posterior temporal lobe made it the most likely region that linked object words with their multiple perceptual semantic representations. This hypothesis still has its strong proponents in an era of imaging white matter tracts in the living human brain by the use of diffusion tensor imaging (Catani and ffytche, 2005). These two authors resurrected the Broca−Wernicke−Lichtheim model, one never abandoned by neurologists. Although Lichtheim had linked speech input (Wernicke's area) to output (Broca's area) via a third region that stored concepts (that is, semantic memories), this was never localized by him to a specific cortical area. Hence, the two eponymous areas of Broca and Wernicke were not accompanied by a third, ‘Lichtheim's area’. Based on Geschwind's hypotheses, Catani and ffytche (2005) placed access to semantic representations, during both speech comprehension and production, in the left inferior parietal cortex, which they labeled as ‘Geschwind's region’.

This role for left inferior parietal cortex has been widely accepted by the clinical neurological community, but based on little direct evidence. The ‘gold standard’ when determining the role of a brain region in cognitive processing is to examine the behavioral consequences of focal lesions, usually infarcts. Strokes confined to the left parietal lobe are rare. For example, Borovsky et al. (2007) performed behavioral analyses on 50 patients with aphasic strokes, but none had lesions confined to the parietal lobe alone.

It was patients with the semantic variant of fronto-temporal lobar degeneration (svFTD) that drew attention to the possibility that it is anterior temporal cortex that provides an amodal route through which semantic representations are accessed (Acosta-Cabronero et al., 2011; Hodges et al., 1992; McClelland and Rogers, 2003; Patterson et al., 2007; Snowden et al., 1989; Warrington, 1975). These patients have a striking and progressive loss of semantic knowledge, irrespective of the modality of stimulus presentation (verbal, environmental sounds, pictures, etc.) (Bozeat et al., 2000). The maximum area of atrophy in these patients is the anterior temporal lobes (ATL), usually with greater atrophy on the left (Acosta-Cabronero et al., 2011). Based on the behavioural sequelae in patients with asymmetrical atrophy, that can manifest as more prominent loss of semantic knowledge in a specific modality (e.g. verbal vs. visual), some have suggested that verbal semantics is more dependent on the left ATL while non-verbal semantics is more dependent on the right ATL (Gainotti, 2012; Mesulam et al., 2013). Others have argued that these findings reflect differences in the strength of connectivity from a bilaterally distributed semantic hub to modality-specific input/output systems. The latter systems for speech production are lateralized to the left hemisphere (Lambon Ralph et al., 2001; Mion et al., 2010). Based on this assertion, it is reasonable to expect that a semantic hub would show functional connectivity with speech ‘output’ systems during retrieval of semantic knowledge while speaking.

As with the inferior parietal lobe, due to vascular anatomy it is rare for the anterior temporal lobe alone to be the location of a stroke. Consequently, lesion-deficit analyses on aphasic stroke patients alone had not identified a role for this region in semantic processing. Furthermore, patients with anterior temporal lobectomy, an operation commonly performed to treat temporal lobe epilepsy, show, at most, only a limited impairment on semantic tasks, the most prominent being anomia (Alpherts et al., 2004; Davies et al., 1995, 2005). This may be because chronic pre-surgical focal epileptic discharges reorganize the normal functional neuroanatomy of the semantic system. An alternative view is that bilateral pathology may be necessary for major impairments of semantic memory (Schapiro et al., 2013); and bilateral temporal lobe surgery for epilepsy has been avoided since lessons from the effects on declarative memory of the consecutive, bilateral medial temporal lobe resections on patient H.M. were revealed. Thus, the argument is that right anterior temporal atrophy contributes to the progressive impairment in svFTD even when the left anterior temporal atrophy is more prominent. However, it could be (and has been) argued that other cortical regions, including parietal cortex, will also have pathological changes, and it may be that diffuse mild atrophy is at least contributing to the semantic deficit. Nevertheless, the same argument would apply to patients with posterior cortical atrophy, a variant of Alzheimer's disease, in whom bilateral occipital, parietal and posterior temporal atrophy is most evident. In addition to their prominent visuo-spatial deficits, these patients develop a progressive linguistic rather than semantic impairment, the reverse of what is observed in patients with svFTD (Crutch et al., 2012, 2013).

Therefore, there are good arguments for a major role for the anterior temporal lobe, or lobes, in semantic processing. Initially, it was hoped that functional neuroimaging results from normal subjects might resolve the debates arising from clinical studies. This has not been realized. Meta-analyses have demonstrated widely distributed cortical regions involved in semantic processing (Binder et al., 2009; Vigneau et al., 2006). Nevertheless, functional magnetic resonance imaging (fMRI) has probably underestimated any contribution from ventral anterior temporal cortex to semantic processing, as signal from this region is lost with conventional fMRI using gradient-echo echo-planar imaging, the consequence of local magnetic field inhomogeneity (susceptibility artifact) (Devlin et al., 2000). Different techniques have been adopted to minimize this effect (Embleton et al., 2010; Halai et al., 2014; Poser and Norris, 2009), with some success. In contrast, functional neuroimaging performed with positron emission tomography (PET), although a more cumbersome and limited technique, can recover signal from this region (Devlin et al., 2000); and PET studies of narrative language comprehension (spoken and written) and narrative speech production, have clearly demonstrated activity in anterior temporal cortex (Awad et al., 2007; Spitsyna et al., 2006). Nevertheless, the ventral AG was also activated in response to narrative language in both these studies, and any differences in function of anterior temporal and inferior parietal cortices could not be determined from the design of these studies.

One possibility is that a component of inferior parietal cortex is part of a system exerting task-dependent control over access to semantic representations, and that activity in this region observed in functional imaging studies of semantic processing reflects this control rather than activation of the semantic system itself (Lambon Ralph, 2014). These proposals have been based on lesion studies, in both stroke patients with ‘semantic aphasia’ (Jefferies, 2006), who have difficulty accessing largely intact semantic representations, and after the induction of temporary partial lesions in normal participants using transcranial magnetic stimulation (Whitney et al., 2011, 2012). It is also a conclusion from the reinterpretation of published functional neuroimaging studies (Noonan et al., 2013). This evidence is further backed up by demonstrations of functional connectivity between left inferior parietal, posterior temporal and posterior frontal cortices during spoken language production (Geranmayeh et al., 2012, 2014), connectivity that is also captured by analyses of ‘rest state’ functional neuroimaging data (Smith et al., 2009).

A further factor that has to be considered is the role of inferior parietal cortex in episodic memory. A review of functional imaging studies that investigated episodic memory retrieval discussed the activity of lateral parietal cortex and the medial retrosplenial and posterior cingulate cortices and the precuneus (Wagner et al., 2005). Studies of narrative speech comprehension and production have consistently demonstrated bilateral AG activity, although often rather more prominent on the left. The narrative tasks depended on retrieval of episodic memories, either personal autobiographic memories or stories that had been illustrated by picture cards prior to scanning that had to be recalled during scanning to elicit narrative speech production (AbdulSabur et al., 2014; Awad et al., 2007; Braun et al., 2001). Listening to narratives results in both encoding and retrieval of episodic information, as the comprehension of an unfolding story only makes sense if later passages can be related to information conveyed earlier in the course of the narrative. In contrast, it can be envisaged that naming or briefly describing a picture weights the task towards semantic memory retrieval and incidental episodic memory encoding, but not on retrieval. Therefore, the dependency on the two forms of declarative memory will be influenced by the specific task used to elicit speech from a participant.

In this fMRI study of overt picture description, we used multivariate analyses of a functional imaging dataset to identify sub-regions within both the left ATL and the left PL. The task required the participants to generate a few facts about the attributes of the object depicted, a task that places little if any explicit demand on episodic memories. However, the pictures, and what the participant chose to say about them, would have been encoded as episodic memories. The functional connectivity of these regions (left ATL and PL) with widely-distributed whole-brain networks that may be engaged during the task were then determined. The ATL signal was optimized, as far as possible, by using a dual-echo acquisition sequence (Halai et al., 2014). In keeping with studies by Lambon Ralph et al. (2001) and Mion et al. (2010), we hypothesized that the left ATL, but not the left PL, would predominantly show connectivity with whole-brain ‘output’ systems known to be strongly activated during task-dependent access to semantics, attributed to the anterior part of Broca's area (Brodmann's area 45) (Thompson-Schill et al., 1997; Wagner et al., 2001), pre-articulatory processes prior to articulation, located to the posterior part of Broca's area (BA44) (Vigneau et al., 2006), and overt articulation itself, located to primary sensory-motor systems (Price, 2012). The functional signature for this system would be significantly greater activity during overt picture description (Speech) when compared with a rest-state baseline (Rest) and two other baseline conditions that were included: counting aloud (Counting) and a ‘yes/no’ decision on simple visual stimuli (Decision).

## 2 Methods

### 2.1 Participants and fMRI procedure

Twenty-five right-handed fluent English-speaking participants (8 male, average age: 57 years, range: 37–78 years) without neurological illness were recruited for the fMRI study. Approval for the study was provided by the National Research Ethics Service Committee-West London.

A Siemens Magnetom Trio 3T scanner was used to derive MRI data using T2*-weighted, gradient-echo, echoplanar, dual-echo parallel imaging (GRAPA) sequence with whole-brain coverage. Thirty-six contiguous axial slices were acquired in an interleaved order (slice thickness, 3 mm; resolution, 3.5×3.5×3.0 mm^3^; field of view, 225×225×108 mm^3^; repetition time (TR), 10 s; acquisition time, 2 s; first echo time (TE1), 13 ms; second echo time (TE2) 31 ms; flip angle, 90°). Quadratic shim gradients were used to correct for magnetic field inhomogeneities within the brain. A high resolution 1 mm^3^ T1-weighted whole-brain structural image, and field maps were also obtained for each subject.

### 2.2 Task fMRI paradigm

The task fMRI was identical to that published in (Geranmayeh et al., 2014). Briefly, a “sparse” fMRI design (Hall et al., 1999) was used to minimize movement- and respiratory-related artifact associated with spoken language production (Geranmayeh et al., 2012; Gracco et al., 2005; Mehta et al., 2006). Tasks were performed in response to specific visual stimuli during an epoch of 7  seconds (s). Following this, a fixation cross was displayed which was the cue for the subjects to discontinue the task. 1 s later whole brain functional imaging data was acquired over 2 s. The cycle was then repeated.

During each scan, the subjects performed three runs of the task. Each run consisted of 71 trials containing 4 conditions; 20 overt picture description (Speech), 16 counting (Count), 16 non-verbal decision response (Decision), and 15 silent rest baseline (Rest). Each condition was pseudo-randomly grouped into blocks of two or four trials.

Of the 25 healthy subjects studied, 22 were scanned again under the same conditions, after a mean interval of 98 days (range 64–173 days) for test retest reliability and as part of a larger ongoing study into aphasic stroke. There was no between session difference in both the BOLD activations, and the in-scanner performances during, the scanning conditions. Therefore to increase the power of this study, the data from both sessions were combined in a mixed effect general linear model.

During the Speech trials, subjects were required to define colored pictures of noun objects selected from a standardized picture set over a 7 s epoch (Rossion and Pourtois, 2004; Snodgrass and Vanderwart, 1980). A total of 120 different pictures representing monosyllabic nouns were displayed at each trial. The participants were instructed to generate as much verbal information pertaining to the given object as possible. The nouns matched across each of the runs and scanning sections with respect to imageability, concreteness, familiarity, and Kucera–Francis frequency based on measures derived from the Medical Research Council psycholinguistic database (Wilson, 1988). As an example of one Speech trial, the participants would view a colored drawing of car and proceed to overtly describe it as “*you drive around in it, it has an engine and four wheels, it is mostly made of metal*”.

During the Count trials, the participants saw a sign “1…” printed in large black font for 7 s, during which time they were required to count up from 1 at a rate of 1/s. During the Rest trials the subjects saw a fixation cross for the entire 8 s before data acquisition. The Decision trials were presented in blocks of four consecutive trials, preceded by a trial containing an instruction page, with a simple written and pictorial instruction, reminding the subjects of the task. The subjects were instructed to press a button placed in the left hand, every time they saw a blue square, and ignore orange circles. During the 7 s, either a blue square or an orange circle was presented at the center of the screen in a random order, each displayed up to a maximum of 1.5 s. The next stimulus followed with a gap of 0.5 s either after 1.5 s had elapsed, or if the subject made a response. The percentage correct responses were calculated. The task itself required no explicit verbal or linguistic processing; audio recording during the Decision task confirmed that less than 0.5% of the decision trials across all subjects involved any overt verbalization.

### 2.3 Behavioral analysis

The speech output was recorded using an MR-compatible microphone (Optoacoustics FOMRI-III noise cancelling microphone). The recordings of Speech trials were transcribed verbatim. The number of appropriate information carrying words (AICW) as defined by the Comprehensive Aphasia Test battery (Swinburn et al., 2005) and the syllables produced per trial were calculated. AICWs are defined as words or word units that convey information, and therefore mainly consist of nouns, verbs, adjectives and adverbs. Any errors of production were excluded in the breakdown of AICWs, but they were included when calculating the rate of syllable production. Fillers (‘um’, ‘er’, etc.) were not included in the count. A Pearson correlation analysis was used to identify the relationship of AICWs to syllables in both runs. These measures were correlated with activations in the brain systems derived from the functional connectivity analysis.

### 2.4 Data preprocessing

Prior to standard preprocessing, fMRI images acquired at the early and late echo times were added together (Halai et al., 2014). This has been shown to improve the signal from the anterior temporal lobes (Halai et al., 2014) which is particularly vulnerable to field inhomogeneities and magnetic susceptibility differences (Devlin et al., 2000). Next, a signal intensity normalization of alternate slices was performed, to correct for the effects of interleaved slice acquisition and 10 s TR, that does not allow for T1 stabilization of spins.

Standard preprocessing was carried out in FMRI Expert Analysis Tool (FEAT) Version 6.00, part of FMRIB's Software Library (FSL, www.fmrib.ox.ac.uk/fsl) in the following manner: motion correction using MCFLIRT (Jenkinson et al., 2002); non-brain voxels removal with Brain Extraction Tool (Smith, 2002); spatial smoothing using a 5 mm full-width half-maximum Gaussian kernel; grand-mean intensity normalization of the entire four dimensional dataset by a single multiplicative factor; and high pass temporal filtering (Gaussian-weighted least-squares straight line fitting, with sigma=50 s) to correct for baseline drifts in the signal. Registration of EPI images to high resolution structural images was carried out by Boundary-Based Registration (Greve and Fischl, 2009) and fieldmap based distortion correction which is expected to further improve the signal from the anterior temporal lobes. The high-resolution structural images were registered to the Montreal Neurological Institute standard space images (MNI 152) using FMRIB's Linear Image Registration Tool (FLIRT). To remove motion related noise, variance associated with six motion variables was removed from the whole brain functional data using ordinary least squares linear regression.

### 2.5 Overview of the connectivity analysis

The outcome of whole brain univariate and multivariate analysis of this study has been published previously (Geranmayeh et al., 2014). In the current paper, we were specifically interested in identifying sub-regions within the left ATL and PL exhibiting functional connectivity with brain systems known to be engaged by the production of propositional speech. We first used a spatially restricted Independent Component Analysis (ICA) to define functional regions of interest (ROI) within the left ATL and left PL. Next a dual regression analysis was performed based on these individual ROIs to identify spatiotemporal signals that matched the time-course of each ROI (for similar methods see Braga et al., 2013; Leech et al., 2012; Simmonds et al., 2014). The spatiotemporal signals (here referred to as systems) that spatially resembled known whole-brain distributed networks that were hypothesized to be activated in the Speech task were identified and their associated subject-specific time courses were regressed against the general linear model design matrices and tested for significance. These will be described in more detail below.

### 2.6 Using a spatially restricted Independent Component Analysis (ICA) to define regions of interest (ROI) within the left ATL and PL

**Fig. 1:**
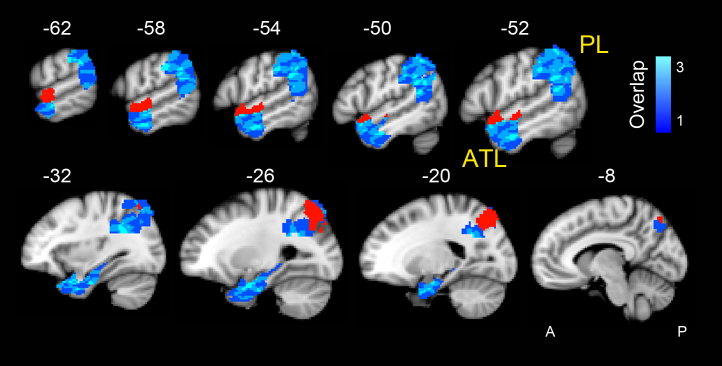
ICA restricted to a left parietal lobe (PL) and a left anterior temporal lo be (ATL) mask resulted in 15 regions of interest (ROI) within each mask. Sagittal T1 weighted slices from the MNI standard space are shown with superimposed ROIs in blue. Different shades of blue show the number of overlapping ROIs. The red ROI within the anterior STS has a unique functional connectivity to a distributed brain system engaged in the Speech task shown in Fig. 2. The red ROI within the superior parietal lobe has a unique functional connectivity with a distributed brain system engaged in the Speech task shown in Fig. 3. Numbers refer to MNI152 atlas coordinates along the *x*-axis. A, anterior; P, posterior. (For interpretation of the references to color in this figure legend, the reader is referred to the web version of this article.)

We first defined *a priori* a spatial mask for the left ATL and PL, and subsequently extracted 15 functional ROIs with separable temporal signals within each mask using an ICA (Fig. 1). For the PL mask, we started with a functionally derived mask of a large parietal lobe region that we have previously shown to be engaged in overt picture description with an independent dataset (region number 3 from Fig. 3 of Geranmayeh et al. (2012)). This was composed of a large lateral parietal region encompassing both the superior and the inferior lobes (supramarginal gyrus and dorsal two thirds of the left angular gyrus (AG)), which in a whole brain ICA, demonstrated functional connectivity with dorsolateral frontal and posterior inferolateral temporal regions during a spoken language production task (Geranmayeh et al., 2012, 2014). In order to have full coverage of the whole inferior parietal lobe, we supplemented this mask with the anatomical mask of the left AG derived from the Harvard-Oxford Cortical structural atlas. Therefore, the final PL mask contained the entire AG, supramarginal gyrus and the superior parietal lobule.

For the ATL, a region not apparent in the whole-brain analysis published by Geranmayeh et al. (2012), the mask was defined anatomically. We combined the left hemisphere anatomical masks available from the Harvard-Oxford cortical structural atlas (http://www.fmrib.ox.ac.uk/fsl/) to create an ATL mask encompassing the temporal pole, anterior portions of the superior temporal, middle temporal, inferior temporal, fusiform and parahippocampal gyri in addition to the entire hippocampus (Fig. 1).

We then performed a spatially-restricted ICA within the PL and ATL masks using group concatenation Probabilistic Independent Component Analysis (Beckmann and Smith, 2004), as implemented in Multivariate Exploratory Linear Decomposition into Independent Components (MELODIC) Version 3.10, part of FSL. The following data pre-processing was further applied to the input data: masking of non-brain voxels, voxel-wise de-meaning, normalization of the voxel-wise variance. Pre-processed data were whitened and projected into a 15-dimensional subspace using Principal Component Analysis. The whitened observations were decomposed into sets of vectors which describe signal variation across the temporal domain (time-courses), the session/subject domain and across the spatial domain (maps) by optimizing for non-Gaussian spatial source distributions using a fixed-point iteration technique (Hyvärinen, 1999). Estimated component maps were divided by the standard deviation of the residual noise and thresholded by fitting a mixture model to the histogram of intensity values (Beckmann and Smith, 2004).

ICA is a multivariate technique that takes advantage of fluctuations in the fMRI data to separate the signal into multiple maximally independent spatiotemporal signals, which may spatially overlap. It has distinct advantages compared to univariate analyses, as it decomposes data in functionally heterogeneous regions, such as the parietal lobe (Cabeza et al., 2012; Humphreys and Lambon Ralph, 2014), where components that respond to the task may overlap anatomically with components of other systems that are either not activated by the task, or even deactivated (Geranmayeh et al., 2014; Seghier et al., 2010). The total variance in the 4D fMRI data is separated amongst the different spatiotemporal components. Each component has a time-course that may relate to a coherent neural signaling associated with a specific task, artifact, or both. For a more detailed explanation of the application of ICA to speech production tasks, see Geranmayeh et al. (2014).

### 2.7 Dual regression analysis*-*functional connectivity with the ROIs from the left ATL and PL

A variant on dual regression was performed on the ROIs that resulted from the ICA decomposition of the PL and ATL. This modified dual regression has been used previously to investigate whole brain connectivity with other brain regions (Leech et al., 2012; Braga et al., 2013; Simmonds et al., 2014). This process involves two regression steps: first, a regression is performed with the 4D fMRI dataset as the dependent variable, and the 15 ROI spatial maps (from the spatially constrained ICA) as the independent variables. For each run for each subject, this first step results in 15 time courses (one for each ROI spatial map). This can be thought of as finding 15 independent temporal signals for each run (each corresponding to a different, but possibly overlapping, ROI). The second regression step again uses the 4D FMRI data as the dependent variable, and the 15 time courses (from the first regression analysis) are the independent variables. This generates 15 statistics for each voxel, assessing how strongly functionally connected the voxel is to each of the ROIs, resulting in 15 whole-brain statistical maps. In this paper we refer to these functional connectivity maps as ‘systems’. These maps were corrected for multiple comparisons using a family wise error rate (*FWE)* of *P*<0.01, *t*>5.6.

### 2.8 Distributed brain systems engaged in the Speech task that were functionally connected to the left PL or ATL

The whole-brain functional connectivity maps of each of the 15 ROIs of the ATL and PL were identified using the dual regression process detailed above. These maps were inspected and excluded from further analyses if: (1) there was a predominant contribution from sources of noise either from movement, CSF, white matter, or variation in head size (Smith et al., 2009); (2) the system was restricted to the cortex immediately surrounding the ROI and/or the homotopic region in the contralateral hemisphere; (3) it resembled components of the default mode network (DMN). The last criterion may appear contentious, as posterior components (lateral inferior parietal cortices and midline cortex posterior to the splenium of the corpus callosum) have been strongly implicated in semantic processes, based on functional neuroimaging results (Binder et al., 2009). However, our previous whole-brain ICA analyses of speech production did not show any components within these regions that were activated by speech; in fact, the reverse, as the posterior components of the DMN were significantly deactivated during picture description (Geranmayeh et al., 2012, 2014). For connectivity maps of regions with functional connectivity with all of the 15 PL and 15 ATL ROIs, see Supplementary Fig. 1. These exclusion criteria resulted in only one ROI from the left ATL, located in the left anterior superior temporal sulcus (antSTS), and one ROI in the left PL, located in the superior parietal lobe (supPL).

Finally, a general linear model was applied to the time course of the system functionally connected to each of these two ROIs, to determine whether the signal was significantly associated with the Speech condition: the run-specific time courses for each subject calculated in the first stage of the dual regression were used as the dependent variable, and the design matrix for the tasks (independent variable) was regressed against them. The resulting *β* coefficient was the estimate of BOLD signal evoked for the different task conditions. The design matrix modeled the time course of Speech, Count, Decision, and Decision Instruction trials at the beginning of each Decision block. Rest trials were the implicit baseline. The run-specific *β* coefficients for each individual were then tested for significance in a mixed effects model (using fitlme in Matlab, with subject modeled as random intercept) in order to identify components where activity was greater during Speech, compared to Rest (Speech>Rest) or Speech contrasted with the higher-level baseline conditions of Count and Decision (Speech>Count+Decision), using one-tailed *t*-test).

### 2.9 Testing the replicability of the whole-brain systems connected to the ATL and PL ROIs

To test the robustness of the two whole-brain systems that connect to the left ATL and PL, we repeated the analysis by varying the dimensionality of the spatially restricted ICA to derive 10 and 25 ROIs within the ATL and PL. The dimensionality of the ICA is usually driven by previous published work, and is chosen somewhat arbitrarily (Smith et al., 2009, 2012). Previous studies using a spatially constrained ICA have used 7, 10, 15 and 24 decomposition (Simmonds et al., 2014; Braga et al., 2013; Leech et al., 2012). Networks defined at lower dimensionalities have, in some cases, been shown to split at higher dimensionality into sub-networks (Smith et al., 2009), whilst higher-dimensional ICA will account for noise more accurately by extracting variations in the data as additional components (Braga et al., 2013).

## 3 Results

### 3.1 Behavioral results

In-scanner speech recording was not available from one subject. For the remaining subjects, across each scanning run, participants spoke 7.42 appropriate information-carrying words (standard deviation or *SD*=1.54) per 7 s trial and 2.64 syllables per second (*SD*=0.59). The two measures significantly correlated with each other (*r*=0.81, *P*<0.0001). There was no significant difference between the two scanning sessions with respect to the syllable rate and appropriate information-carrying words (paired *t-*test, *P>*0.05).

During the Decision task, subjects correctly identified the blue square target on 98.8% of trials, and correctly inhibited a response to the orange circles on 99% of trials, indicating a ceiling effect with respect to task difficulty. During the Count trials the participants spoke 7.17 words per 7 s trial (*SD*=1.5).

### 3.2 Whole brain systems engaged in Speech that functionally connect with the ATL

**Fig. 2:**
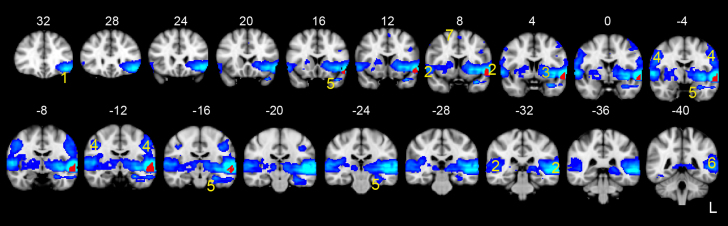
The distributed whole-brain system in blue, is functionally connected with the left anterior superior temporal sulcus (antSTS) in red. Activity in this system showed a positive correlation with behavioral measures of speech production during the scan. Activity is overlaid on standard T1-weighted anatomical slices, *FWE* corrected *P*<0.01, *t*>5.6. Numbers above the slices refer to MNI152 atlas coordinates along the *y*-axis. The numbered regions are: 1, left inferior frontal gyrus including pars triangularis and opercularis; 2, bilateral primary and association auditory cortices; 3, left more than right insular activity extending into putamen; 4, bilateral sensory-motor cortices; 5, left hippocampus and parahippocampus extending into the anterior temporal fusiform cortex. 6, on the left the superior temporal cortex activity borders the most ventral part of the inferior parietal lobe; 7, small amount of activity is also seen in supplementary motor cortex. See Table 1 for peak coordinates. (For interpretation of the references to color in this figure legend, the reader is referred to the web version of this article.)

We first set out to identify whole brain systems that functionally connected with one or more of the 15 anterior temporal lobe ROIs and that may be engaged in semantic retrieval when verbally describing objects. These ROIs are shown in Fig. 1 (with the two mask regions shown in blue and the specific ROIs shown in red). As described in the methods only one ROI in the ATL and one in the PL mask matched the criteria for further evaluation. The red ATL ROI in Fig. 1 is located in the anterior superior temporal sulcus (antSTS). This was functionally connected to the distributed whole-brain system shown in Fig. 2 (also see Table 1).

This system included activity in: a large left inferior frontal gyrus region, including pars triangularis and opercularis; bilateral primary and association auditory cortices, including planum temporale and Heschel's gyrus; left more than right insular activity extending into putamen; bilateral primary sensory-motor cortices. A small, but statistically significant, region of activity was also seen in the supplementary motor area. Other temporal lobe regions included the left hippocampus and parahippocampal gyrus, extending into temporal fusiform cortex. There was no activity in the right temporal cortex. The activity in the left posterior superior temporal cortex merged with the most ventral part of the left PL.

A mixed-effect analysis confirmed that activity in this network was significantly greater during the contrast of Speech against the baseline conditions (Speech>Rest: *t*=1.8, *P*=0.04; Speech>Count+Decision: *t*=2.0, *P*=0.025). In addition, the activity in this system, showed a significant positive relationship with both the average number of AICW (*t*=1.76, *P*=0.04, 1-tailed, 43 DF) and the syllable rate (*t*=2.08, *P*=0.02, 1-tailed, 43 DF), although there was no significant relationship between either variable with activity, when partialling out the effect of the other. This is not surprising as syllable rate and AICW are not orthogonal in normal subjects. Inevitably the analysis of such a task may be unable to separate semantic from sensory (auditory and somatosensory) and motor processes associated with overt speech production. On the other hand this system was significantly more active for Speech than the higher-level baseline tasks of Counting and Decision (both requiring sensory-motor responses to execute the task, and in the case of Counting with the same word production rate as in Speech (*P*=0.40).

### 3.3 Whole brain systems engaged in Speech that functionally connect with the PL

**Fig. 3:**
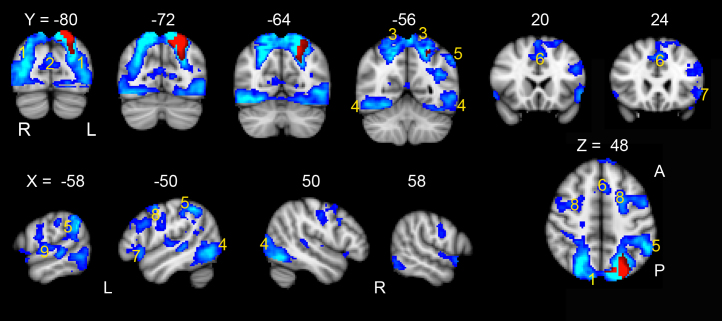
The distributed whole-brain system in blue, is functionally connected with the left superior parietal lobe (supPL) in red. Activity in this system was significantly related to the Speech task compared to the higher-level baseline Count and Decision tasks. Activity is overlaid on standard T1-weighted anatomical slices, *FWE* corrected *P*<0.05, *t*>5.6. Numbers above the coronal slices refer to MNI152 atlas coordinates along the *y*-axis and those above sagittal slices refer to coordinates along the *x*-axis. One axial slice is shown at *z*=48 mm. The numbered regions are: 1 bilateral lateral occipital cortices 2 cuneal cortices, 3 superior parietal lobes 4 occipital poles extending anteriorly to posterior fusiform gyrus/lingual gyrus, 5 left angular gyrus and posterior supramarginal gyrus, 6 left superior frontal gyrus extending into dorsal anterior cingulate, 7 posterior inferior frontal gyrus, 8 superior frontal sulcus extending into middle frontal gyrus and inferior frontal sulcus 9 left lateralized posterior superior temporal gyrus. A, anterior; P, posterior. See Table 2 for peak coordinates. (For interpretation of the references to color in this figure legend, the reader is referred to the web version of this article.)

The ROI in the PL (Fig. 1) was located in the superior parietal lobe (supPL). This ROI was functionally connected to the distributed whole brain system shown in Fig. 3. This system predominantly incorporated regions known to be involved in processing of the visual stimuli. Posterior areas of activity related to visual processing included: bilateral lateral occipital cortices, extending superiorly to superior parietal lobe; bilateral posterior midline cortices (the cuneus) and visual cortices extending anteriorly to the posterior fusiform/lingual gyri. Some of these regions are known to be part of the dorsal attention network (DAN) that has been linked to attentional processing of visual stimuli (Corbetta and Shulman, 2002; Braga et al., 2013). Activity was also observed in the frontal eye fields in the left and right posterior superior frontal sulci. In addition, there was activity in the dorsal anterior cingulate gyrus and adjacent superior frontal gyrus, with activity also in bilateral posterior inferior frontal gyri. This system, known as the cingulo-opercular system or salience network (Dosenbach et al., 2008; Seeley et al., 2007), is a frontal executive system involved in cognitive control. There was a large cluster of activity in the left posterior supramarginal gyrus extending to the inferior AG. Additional small clusters of activity were observed in the bilateral superior temporal gyri (left more than right), and in pre- and post-central gyri (see Table 2).

A mixed-effect analysis confirmed that activity in this network was significantly more during the contrast of Speech against higher-level baseline conditions (Speech>Count+Decision; *t*=3.6, *P*<0.001). There was no correlation of activity in this system with measures of speech production, namely AICW or syllable rate (*P*>0.5).

Specifically, none of the ROIs that at least partially overlapped with the AG mask from the Harvard-Oxford Cortical structural atlas (Supplementary Fig. 1, PL ROIs: 2, 6, 7, 8, 9, 10, 11, 14), showed connectivity with systems that were significantly more activated during Speech.

### 3.4 Robustness of the brain systems connected to the ATL and PL

ICA splits up the variance in the fMRI data into a number of spatially separable but possibly partially overlapping components. The number of these components is dependent on the specified dimensionality of the ICA. Setting a higher dimensionality in ICA decomposes larger networks into more discrete sub-networks (Smith et al., 2009). In order to test the stability of the two systems that connect to the antSTS and supPL respectively, we performed the spatially restricted ICA on the ATL and PL at a lower (10) and a higher (25) dimensionality.

**Fig. 4:**
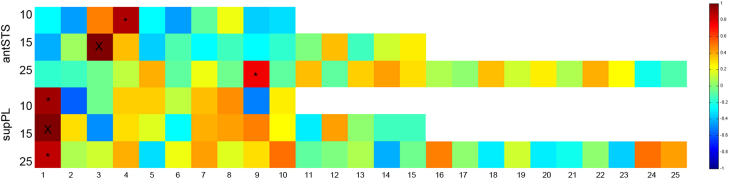
Correlation matrix showing the pairwise spatial correlation of networks derived from dual-regression analyses. Top three rows show the spatial correlation between the system shown in Fig. 2 (blue) and all the networks linked to the ATL ROIs derived from 10, 15 and 25 decomposition of the ATL. Bottom three rows show the spatial correlation between the system shown in Fig. 3 (green) and all the networks linked to PL ROIs derived from 10, 15 and 25 decomposition of the PL. The numbers on the *y*-axis denote the dimensionality of the ICA used to derive the ROIs. The color bar refers to the correlation coefficient. Blue colors have a low correlation coefficient; red colors have a high correlation coefficient. “*X*” denotes the correlation of the systems in Figs. 2 and 3 with themselves (*r*=1). The top two “*” refer to the networks that connect to the left antSTS and have the highest spatial correlation to the system in Fig. 2. The bottom two “*” refer to the networks that connect to the left supPL and have the highest spatial correlation to the system in Figs. 3. These analyses suggest that the systems identified in Figs. 2 and 3 are spatially robust. (For interpretation of the references to color in this figure legend, the reader is referred to the web version of this article.)

For the ATL ROIs, a system connecting with the antSTS and with a high spatial correlation to that shown in Fig. 2 was identified at each dimensionality (*r*>0.88 for 10 ROIs and *r*>0.75 for 25 ROIs) (Fig. 4, top three rows). For the 25 component decomposition of the left ATL, the time course of this system remained significantly associated with the Speech trials (Speech>Rest: *t*=3.0, *P*=0.02; Speech>Count+Decision: *t*=1.8, *P*=0.04). A similar trend was identified when decomposing the left ATL to 10 ROIs although it failed to reach significance (Speech>Rest: *t*=1.2, *P*=0.12; Speech>Count+Decision: *t*=1.18, *P*=0.12).

With regards to the PL ROIs, a system connecting with the left supPL and with a high spatial correlation to that shown in Fig. 3 was identified at each dimensionality (*r*>0.93 for 10 ROIs and *r*>0.86 for 25 ROIs) (Fig. 4, bottom three rows). For both the 10 and 25 component decomposition of the left PL, the time course of this system remained significantly associated with the Speech trials compared to high-level baselines (10 ROIs Speech>Count+Decision: *t*=2.83, *P*=0.007. 25 ROIs Speech>Count+Decision: *t*=3.92, *P*<0.001).

We also performed the analysis by deriving the ROIs from a resting state data set (see Supplementary material) and found qualitatively similar results. These analyses suggest the systems illustrated in Figs. 2 and 3 are spatially robust, and connected to the antSTS and supPL respectively.

## 4 Discussion

The speech production task involved the participants viewing a colored-drawing of an object while selecting, under time pressure, from amongst the many items of semantic knowledge that they possessed about that object, before translating that selection into a few overt phrases. At the end of this period, lasting 7 s, a whole-brain functional image was acquired. Therefore, the recorded BOLD signal will have contained activity associated with many processes, from early visual processing through to the sensory-motor processes involved in articulation. This will have included semantic and linguistic processes, systems involved in attention and domain-general cognitive control (Hampshire et al., 2012; Duncan 2010); and those engaged when making selections from among competing alternative semantically related responses, (Thompson-Schill et al., 1997; Wagner et al., 2001).

The purpose was to identify from these many activated systems the one or a few by which attributes were selected from semantic memory and processed through to speech production. In terms of topography, the study was designed to demonstrate whether one or more components in either the left PL or ATL were functionally connected to the left inferior frontal gyrus (known to be activated during selection of competing semantic responses) and the sensory-motor cortices responsible for the overt expression of those response.

The functional connectivity results supported the hypothesis that it was a component of the left ATL, namely the antSTS, that plausibly connects the proposed semantic hub in anterior temporal cortex with selection signals from the left inferior frontal gyrus, coupled with signal that encompassed the frontal operculum and bilateral sensory-motor cortices responsible for pre-articulatory and articulatory processes, respectively. Additional activity in bilateral primary and association auditory cortices is explained by the response to the sound of overt speech production.

It is evident that this component conflated a number of different cognitive, motor and sensory processes. Nevertheless, the result is compatible with the hypothesis that a semantic hub, located in ventral ATL, is functionally connected via the ant STS with the inferior frontal gyrus. The connections will be reciprocal. It has been proposed that amodal semantic processing takes place in bilateral ATL (Patterson et al., 2007) and more specifically the ventral ATL (Binney et al., 2010; Mion et al., 2010; Sharp et al., 2004; Visser and Lambon Ralph, 2011; Visser et al., 2012), but only a left-lateralised response in the ventromedial ATL was observed in this study; compatible with the notion that lateralization is imposed by the ‘output’.

Therefore, based on this result we propose that the anterior STS, a subcomponent of the ATL, connects the left ventromedial ATL to the inferior frontal gyrus during the performance of a verbal semantic task based on a visual stimulus. This pathway has been promoted as the ‘ventral’ language pathway, the consequence of studies in both non-human primates (e.g. Romanski et al., 1999) and humans (e.g. Saur et al., 2008), reviewed by Rauschecker and Scott (2009). The dorsal bank of the STS is recognized as multisensory cortex, both in primates (Padberg et al., 2003) and in humans (Beauchamp et al., 2008). Schmahmann et al. (2007) used combined tracer techniques and diffusion spectrum imaging on monkey brains, and demonstrated that the STS is connected to BA 45 and 47 via the extreme capsule. Further evidence for the structural connectivity between the ventral ATL and inferior frontal gyrus via the antSTS, comes from an in vivo human tractography study by Binney et al. (2012). Despite considerable intra-temporal connectivity within the let temporal lobe, the study failed to identify a direct white matter tract between the ventral ATL and Broca's area. Instead, superior temporal gyrus (including the antSTS) showed considerable connectivity to both ATL and inferior frontal gyrus. In all probability, components of the left IFG that showed strong connectivity with the ATL, mediated multiple functions including both domain-specific (encoding the retrieved semantic attributes as verbal messages) and domain-general executive control involved in selection of responses (Thompson-Schill et al., 1997; Fedorenko et al., 2012; Wagner et al., 2001).

**Fig. 5:**
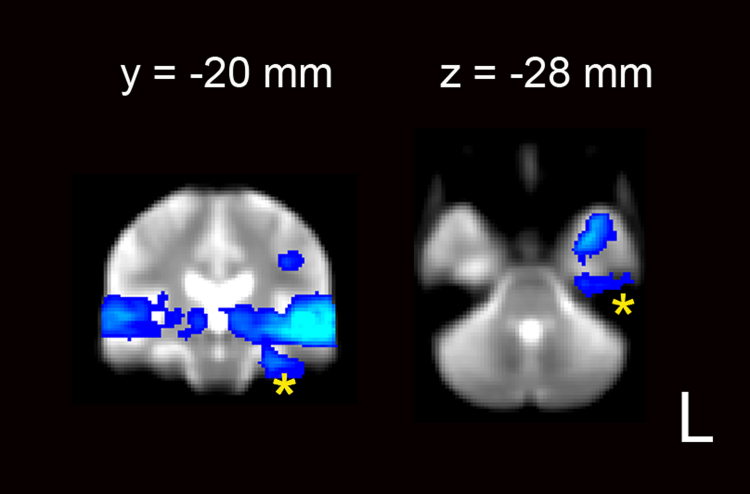
Persistent susceptibility artifact in the inferior-medial ATL despite measures to minimize this effect. Activity from Fig. 2 is superimposed on the mean functional image from the study transformed into the standard MNI space. One coronal and one axial slice are shown at the MNI coordinates specified. The activity in the parahippocampus and fusiform cortex in blue extends into the edge of the susceptibility artifact as shown with an asterix. The activation is *FWE* corrected at *P*>0.01, *t*>5.6.

Based on PET metabolic imaging in patients with svFTD, Mion et al. (2010) proposed that the anterior fusiform gyrus, probably incorporating a lateral extension of perirhinal cortex (a heteromodal region strongly connected to third order sensory association cortices), constitutes the semantic ‘hub’. Perirhinal cortex has a central role in object association memory in the monkey (e.g. Hirabayashi et al., 2013). This ATL region is precisely where it is difficult to recover signal because of susceptibility artifact, accounting for the relatively weak, and truncated, signal from the ventral ATL in this (and many other) fMRI studies (Fig. 5). On the assumption that there was, in reality, much greater activity in this region, then the results from this study can be interpreted as demonstrating functional connections between ventral ATL including perirhinal cortex, polysensory antSTS, and, via the extreme capsule, the left inferior frontal gyrus. Thus the weakened ventral ATL signal from an ROI (ICA component) localized to this region may not have been able to capture its whole brain connectivity. Instead, the ROI based on the antSTS was able to show connectivity with both the inferior frontal and ventral ATL regions.

In addition to the evidence gathered from studies of patients with svFTD, referred to in the introduction, evidence form several other techniques support the contribution of the ATL to semantic processing. First, voxel-based lesion-symptom mapping in patients with post-stroke aphasia, has implicated the left ATL in naming and semantic deficits (Butler et al., 2014; Schwartz et al., 2009, 2011). Second, repetitive transcranial magnetic stimulation (rTMS) over the lateral ATL, has been shown to mirror picture naming deficits observed in svFTD, in neurologically intact participants (Pobric et al., 2007, 2010). Third, neurophysiological recordings from intracranial electrodes in patients with epilepsy have implicated the ventral ATL in picture naming (Lüders et al., 1986; 1991) and semantic processing (Nobre and McCarthy, 1995; Nobre et al., 1994; Shimotake et al., 2014).

In contrast to the ATL, one component of the left parietal lobe, activated specifically by the Speech condition and located in the superior parietal lobe (supPL), was functionally connected to systems known to be predominantly engaged in recognising, scanning, and focusing attention on the pictorial stimuli. Activity was observed in bilateral ventral temporal cortex, responsible for the processing of the visual features of the coloured pictures, the perceptual ‘input’ system, and dorsal regions comprising those responsible for exploratory eye movements and the dorsal attention network (Corbetta and Shulman, 2002; Braga et al., 2013). There was additional co-activation of the left AG and left inferior frontal sulcus, which were connected to this predominantly ‘input’ processing system. In contrast to the connectivity of the antSTS, there was only restricted regions activated in bilateral sensory-motor and premotor cortices and bilateral inferior frontal gyri. No ROIs localized entirely or in part with the left AG were activated solely by the Speech condition.

The activity in the AG and inferior frontal sulcus are consistent with the proposed involvement of these regions in cognitive control (Duncan, 2010; Erika–Florence et al., 2014; Geranmayeh et al., 2014) and more specifically semantic control (Lambon Ralph, 2014; Noonan et al., 2013) required in the Speech task. Importantly, unlike the ‘output’ system connected to the left antSTS, the activity in this predominantly ‘input’ system did not correlate with the number of AICW produced for each picture. The AG is a functionally heterogeneous region and has been implicated in many domains other than semantics (Cabeza et al., 2008, 2012; Corbetta and Shulman, 2002; Humphreys and Lambon Ralph, 2014). Humphries et al. (2014) proposed a dorsal vs. ventral division within the parietal lobe with the dorsal regions (superior parietal lobe and intraparietal sulcus) being associated with executive-demand processes such as top-down attention, and phonological and semantic decisions. More ventrally the AG was associated with automatic tasks, episodic memory retrieval, sentence level processing as well as the DMN, whilst the supramaginal gyrus was associated with bottom-up attentional processes.

Therefore, the analysis was able to separate these two very broad ‘input’ (with connectivity to the PL) and ‘output’ (with connectivity to the ATL) systems, despite the fMRI signal being obtained after both systems had become active. The use of temporally slow ‘sparse’ sampling in this way was necessary because of the unacceptable noise associated with overt articulation of connected speech. This prevented the use of what would otherwise have been the preferred method, in which rapid event-related functional imaging is used to sample signal weighted towards different time points over the time course of initial stimulus presentation through to response.

## Figures and Tables

**Table 1:** Coordinates for each local maxima within significant clusters of activity of the system shown in Fig. 2 (*FWE* corrected *P*<0.01, *t*>5.6). *L*, left; *R*, right.

Cluster size (voxels)	Cluster size (cm3)	*t*	MNI coordinate at maximum *t* (*x y z*)			Harvard-Oxford Cortical structural atlas
23,875	191	25.3	−54	8	−10	L temporal pole/L anterior superior temporal gyrus extending to L parahippocampal gyrus, L inferior frontal gyrus (pars opercularis and pars triangularis), bilateral auditory cortices and sensory-motor cortices
56	0.45	7.07	−42	10	30	L middle frontal gyrus, precentral gyrus
28	0.22	6.98	0	10	60	Supplementary motor cortex
1	0.01	5.67	−2	0	38	Anterior cingulate cortex
1070	8.56	8.69	46	−82	−2	Inferior division of R lateral occipital cortex
68	0.54	6.6	−28	−96	4	Inferior division of L lateral occipital cortex

**Table 2:** Coordinates for each local maxima within significant clusters of activity of the system shown in Fig. 3 (*FWE* corrected *P*<0.01, *t*>5.6). *L*, left; *R*, right.

Cluster size (voxels)	Cluster size (cm^3^)	*t*	MNI coordinate at maximum *t* (*x y z*)			Harvard-Oxford Cortical structural atlas
31,150	249.2	23.1	−16	−72	54	L superior lateral occipital cortex extending into superior lateral occipital cortex
Local maxima		14.7	−56	−52	40	L posterior supramarginal gyrus. L angular gyrus
		9.88	−38	−44	66	L superior parietal lobule
		11	−40	8	56	L middle frontal gyrus extending to superior frontal sulcus and gyrus
		9.55	−46	12	30	
		9.5	−52	20	-6	L posterior inferior frontal gyrus
		9.59	−66	−18	4	L posterior superior temporal gyrus
		9.89	−48	−36	2	
		10.3	0	14	60	Superior frontal gyrus
		9.17	−2	22	54	Dorsal anterior cingulate
		10.6	−4	−86	36	L Cuneal cortex
		9.15	2	−80	26	R Cuneal cortex
		8.68	−4	−98	2	L occipital pole extending into lingual gyrus
		10.2	−10	−30	−2	Left thalamus
1400	11.2	8.71	32	−2	46	R superior frontal sulcus
100	0.8	7.3	46	−34	8	R posterior superior temporal gyrus
37	0.296	6.38	22	−22	52	R precentral gyrus
35	0.28	6.27	−22	−30	54	L precentral gyrus
