# Supplementary material for: Semantic retrieval during overt picture description: Left anterior temporal or the parietal lobe?
Source: Neuropsychologia. 2015 Sep;76:125–35. doi: 10.1016/j.neuropsychologia.2014.12.012 (PMC4582804; doi:10.1016/j.neuropsychologia.2014.12.012)
Supplement: Supplementary file 1 — Supplementary material. [file mmc1.docx]

**Supplementary Material 1**

This figure shows the functional connectivity maps (red/yellow) of each of the 15 ROIs (green) derived from the left ATL and PL masks. Activity of the connectivity map is overlaid on standard T1-weighted anatomical slices that best represented the distribution of the map, *FWE* corrected *p* < 0.05, *t* > 5.6. Numbers above the axial slices refer to MNI152 atlas coordinates along the z-axis. Numbers along the slices refer to the order of ROIs derived when performing spatially restricted ICA on the ATL and PL masks. The distributed brain systems marked with * were taken forward for further analysis. The remainder were excluded if they satisfied any of the following criteria: 1) there was a predominant contribution from sources of noise either from movement, CSF, white matter, or variation in head size (Smith *et al*., 2009); 2) the system was restricted to the cortex immediately surrounding the ROI and/or the homotopic region in the contralateral hemisphere; 3) it resembled components of the default mode network (DMN). The DMN-like components had high spatial correlation (r > 0.43) with the DMN map published by Smith and colleagues (2009). The later criterion was included as the DMN has been shown to be deactivated during fMRI studies of overt speech production (Geranmayeh *et al*. 2012; 2014; Seghier *et al.*, 2010) using similar time-limited tasks to the current study.

**Supplementary Material 2**

We repeated the analysis by deriving the PL and ATL ROIs, from a different fMRI data set. This data set consisted of resting state data from 46 right-handed fluent English-speaking participants, 23 of whom underwent the task fMRI as well. Of these twenty-three two resting state scans were available in 21 subjects. An additional 23 subjects also underwent the same resting state protocol only once. The acquisition parameters for the resting state fMRI were identical to that of the task fMRI, except that 180 volumes were acquired with a TR of 2s. The subjects were instructed to close their eyes but not fall asleep during this time.

A spatially similar network to that shown in figure 2 and connecting to the antSTS, was identified irrespective of the number of ROIs in the ATL (see top three rows of the supplementary figure, denoted by * ). For the 15 and 25 decomposition of the ATL, the time course of the matching networks (denoted by “ * ”) were associated with the Speech task and a similar trend was identified when decomposing the ATL to 10 ROIs. (10 ROI: Speech > Rest, *t* = 2.0 *P* = 0.03; Speech > Count + Decision, *t* = 1.49 *P* = 0.07. 15 ROI: Speech > Rest, *t* = 2.4 *P* = 0.01; Speech > Count + Decision, *t* = 1.32, *P* = 0.09. 25 ROI: Speech > Rest, *t* = 3.2, *P* = 0.001; Speech > Count + Decision, *t* = 2.3, *P* = 0.01 ).

A spatially similar network to that shown in figure 3 and connecting to the supPL, was identified irrespective of the number of ROIs in the PL (see bottom three rows of the supplementary figure, denoted by * ). For all the decomposition of the PL, the time course of the matching networks (denoted by “ * ”) were more significantly associated with the Speech task compared to Count and Decision baseline (Speech > Count + Decision: 10 ROI *t* = 1.9 *P* = 0.04. 15 ROI *t* = 5.0, *P* < 0.001. 25 ROI *t* =11.9, *P* < 0.001).

**Supplementary figure 2.** Correlation matrix showing the pairwise spatial correlation of networks derived from dual-regression analysis. Top three rows show the spatial correlation between the system shown in figure 2 and all the networks functionally linked to left ATL ROIs derived from 10, 15 and 25 decomposition of the left ATL of the resting state data. Bottom three rows show the spatial correlation between the system shown in figure 3 and all the networks linked to left PL ROIs derived from 10, 15 and 25 decomposition of the PL of the resting state data. The numbers on the Y axis denote the dimensionality of the ICA used to derive the ROIs. The color bar, refers to the correlation coefficient. Blue colors have a low correlation coefficient; red colors have a high correlation coefficient. The top three “ * ” refer to the networks connecting to antSTS and with the highest spatial correlation to the system in figure 2 (*r* = >0.62). The bottom three “ * ” refer to the networks connecting to supPL and with the highest spatial correlation to the system in figure 3 (*r* > 0.56). These analyses suggest that the systems identified in figure 2 and also 3 are spatially robust.
