# Supplementary figures and images for: Semantic retrieval during overt picture description: Left anterior temporal or the parietal lobe?
Source: Neuropsychologia. 2015 Sep;76:125–35. doi: 10.1016/j.neuropsychologia.2014.12.012 (PMC4582804; doi:10.1016/j.neuropsychologia.2014.12.012)

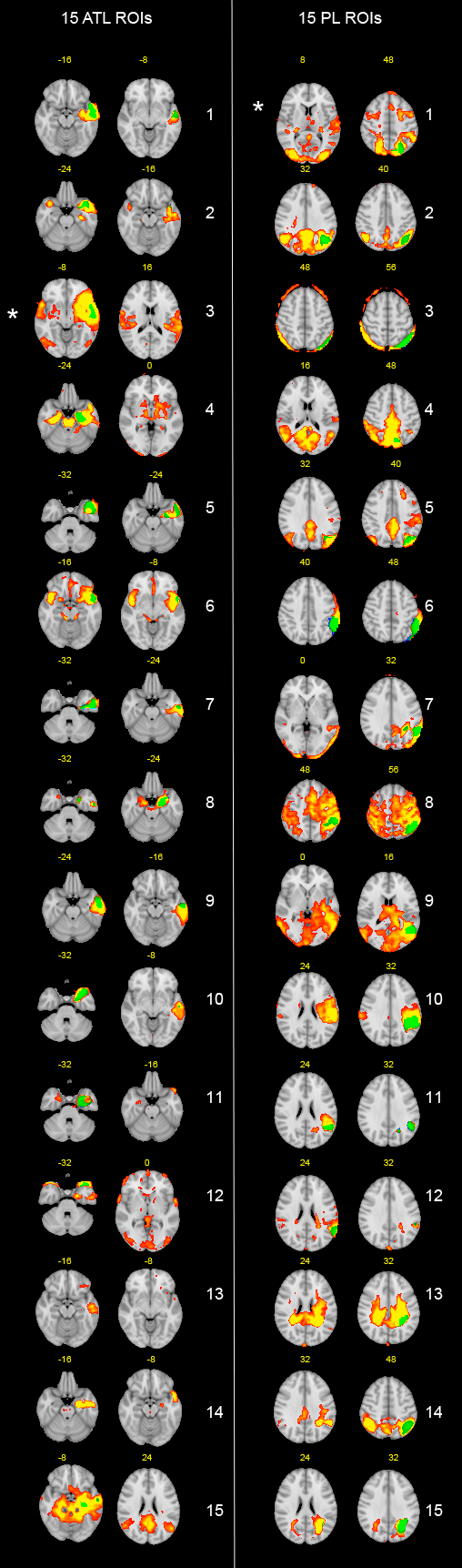

Supplement: Supplementary file 2 — Supplementary material. [file mmc2.zip › Supplement_fig1.tif]

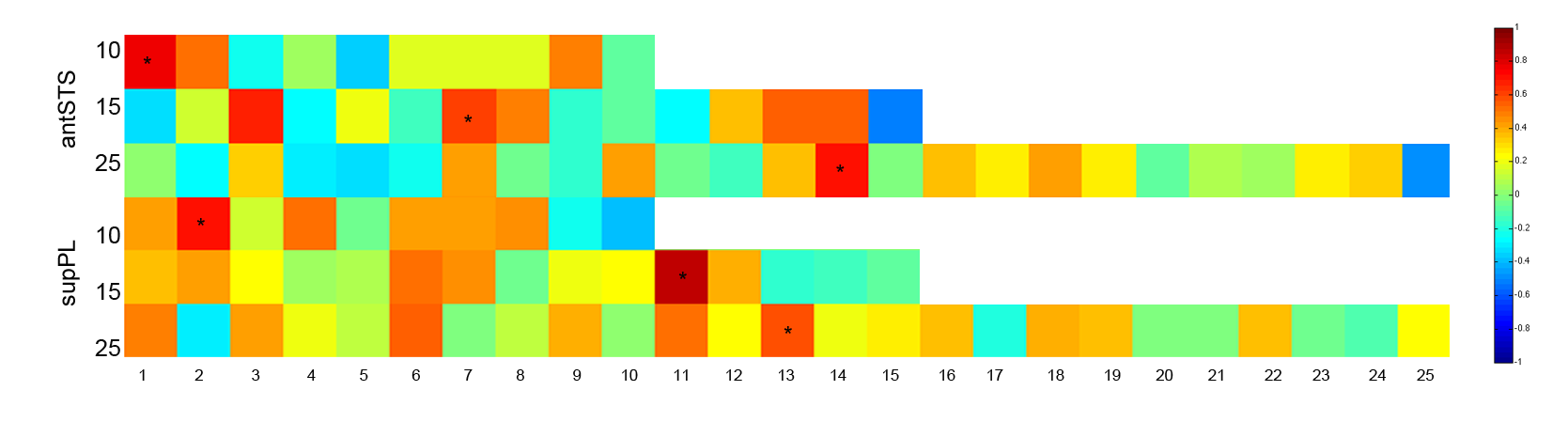

Supplement: Supplementary file 3 — Supplementary material. [file mmc3.zip › supplementary_Figure2.tif]
